# Supplementary material for: Developmental trajectories of EEG aperiodic and periodic components in children 2–44 months of age
Source: Nat Commun. 2024 Jul 10;15:5788. doi: 10.1038/s41467-024-50204-4 (PMC11237135; doi:10.1038/s41467-024-50204-4)
Supplement: Supplementary file 3 — Reporting Summary [file 41467_2024_50204_MOESM3_ESM.pdf]

nature portfolio

Corresponding author(s): Carol Wilkinson

Last updated by author(s): Jun 13, 2024

Reporting Summary

Nature Portfolio wishes to improve the reproducibility of the work that we publish. This form provides structure for consistency and transparency in reporting. For further information on Nature Portfolio policies, see our Editorial Policies and the Editorial Policy Checklist.

Statistics

For all statistical analyses, confirm that the following items are present in the figure legend, table legend, main text, or Methods section.

n/a	Confirmed	
☐	☒	The exact sample size (*n*) for each experimental group/condition, given as a discrete number and unit of measurement
☐	☒	A statement on whether measurements were taken from distinct samples or whether the same sample was measured repeatedly
☐	☒	The statistical test(s) used AND whether they are one- or two-sided<br>*Only common tests should be described solely by name; describe more complex techniques in the Methods section.*
☐	☒	A description of all covariates tested
☐	☒	A description of any assumptions or corrections, such as tests of normality and adjustment for multiple comparisons
☐	☒	A full description of the statistical parameters including central tendency (e.g. means) or other basic estimates (e.g. regression coefficient) AND variation (e.g. standard deviation) or associated estimates of uncertainty (e.g. confidence intervals)
☐	☒	For null hypothesis testing, the test statistic (e.g. *F*, *t*, *r*) with confidence intervals, effect sizes, degrees of freedom and *P* value noted<br>*Give P values as exact values whenever suitable.*
☒	☐	For Bayesian analysis, information on the choice of priors and Markov chain Monte Carlo settings
☒	☐	For hierarchical and complex designs, identification of the appropriate level for tests and full reporting of outcomes
☒	☐	Estimates of effect sizes (e.g. Cohen's *d*, Pearson's *r*), indicating how they were calculated

Our web collection on statistics for biologists contains articles on many of the points above.

Software and code

Policy information about availability of computer code

Data collection	Not applicable
Data analysis	EEG Processing utilized publicly a available pipeline run via MATLAB: (1) Batch Automated Processing Platform (BEAPP) and the Harvard Automated Preprocessing Pipeline for EEG. A modified version of FOOOF v1.0.0 was also used and code made available. Analyses and figures were completed using Python v3.6.8 or in R (version 4.1.2). BEAPP/HAPPE inputs and analysis code is available here: https://osf.io/u3gp4

For manuscripts utilizing custom algorithms or software that are central to the research but not yet described in published literature, software must be made available to editors and reviewers. We strongly encourage code deposition in a community repository (e.g. GitHub). See the Nature Portfolio guidelines for submitting code & software for further information.

Data

Policy information about availability of data

All manuscripts must include a data availability statement. This statement should provide the following information, where applicable:

- Accession codes, unique identifiers, or web links for publicly available datasets
- A description of any restrictions on data availability
- For clinical datasets or third party data, please ensure that the statement adheres to our policy

Consents obtained from human participants prohibit sharing of de-identified individual data without data use agreement in place. Please contact the corresponding author with reasonable data requests.

More detail: The current longitudinal analysis uses data that were collected across various independent projects. Because of this, our ability to share data depends on several factors: which specific data are requested; whether and to what extent the participants included in the requested data have consented to the sharing and future use of their data; whether deidentified, limited, or identified data are requested; and the purpose for which the data are requested. Prior to sharing data, we may need to confirm whether Boston Children's Hospital and the institution of the individual requesting the data have existing agreements or subcontracts with terms of data use and sharing that define the collaboration (e.g., data use agreements, data access agreements, subawards, reliance agreements).

Research involving human participants, their data, or biological material

Policy information about studies with human participants or human data. See also policy information about sex, gender (identity/presentation), and sexual orientation and race, ethnicity and racism.

Reporting on sex and gender	This study uses the term sex, to describe the sex assigned at birth, as participants were enrolled prior to 12 months of age.
Reporting on race, ethnicity, or other socially relevant groupings	Race and ethnicity is provided for the sample to provide readers with an understanding of the degree of diversity of the sample. This information was obtained from the parent of the participant when completing a demographics survey specifically asking about their child's ethnicity and race. Mixed race is used to describe participants who parents identify with multiple racial groups.
Population characteristics	This is a longitudinal data set across infants from 2-44 months old and therefore age was used in analyses of longitudinal trajectories. Sex was also used as a covariate to determine whether there were differences in trajectories based on sex. Since data came from different studies, this was also included as a covariate.
Recruitment	Please see the manuscript for details. For the longitudinal trajectory portion of this paper, participants were recruited through a hospital research registry, direct recruitment from a primary care clinic, as well as social media advertisements. Bias can exist in who is able to participate in research, as not families must be aware of the study to participate, and must have the time and resources to be able to come to multiple 3-4 hour study visits.
Ethics oversight	Boston Children's Hospital for studies analyzed as part of longitudinal trajectories. Montefiore Medical Center for analyses of EEG collected from patients receiving anesthesia.

Note that full information on the approval of the study protocol must also be provided in the manuscript.

Field-specific reporting

Please select the one below that is the best fit for your research. If you are not sure, read the appropriate sections before making your selection.

[x] Life sciences [ ] Behavioural & social sciences [ ] Ecological, evolutionary & environmental sciences

For a reference copy of the document with all sections, see nature.com/documents/nr-reporting-summary-flat.pdf

Life sciences study design

All studies must disclose on these points even when the disclosure is negative.

Sample size	No sample sizes were determined for this analysis. Data from 4 longitudinal studies were combine resulting in a large sample size - a sample much larger than previously published studies.
Data exclusions	Longitudinal analysis: All infants had a minimal gestational age of 36 weeks, no history of prenatal or postnatal medical or neurological problems, and no known genetic disorders. Infants who were later diagnosed with ASD (either by assessment during the study, or by community diagnosis disclosed by parents prior to age of 5) were not included in this study. This exclusion was performed into order to limit the analysis to healthy infants without known reasons to have atypical neurodevelopment. For the anesthesia cohort, we similarly aimed to limit the analysis to healthy infants and therefore infants were excluded for prematurity, known neurologic injury, epilepsy, or planned intracranial surgery.
Replication	Outside of the analyses presented in the paper, we qualitatively assessed consistency in age-dependent changes in aperiodic and periodic power spectra between the 4 different studies and confirmed that identified changes are observed across all 4 studies.
Randomization	There was no randomization for this analysis.
Blinding	Analysis pipelines were used for artifact removal that are automated and reproducible. This prevents biases that can occur with artifact-removal processes dependent on human decisions.

Reporting for specific materials, systems and methods

We require information from authors about some types of materials, experimental systems and methods used in many studies. Here, indicate whether each material, system or method listed is relevant to your study. If you are not sure if a list item applies to your research, read the appropriate section before selecting a response.

Materials & experimental systems

n/a	Involved in the study
☒	☐ Antibodies
☒	☐ Eukaryotic cell lines
☒	☐ Palaeontology and archaeology
☒	☐ Animals and other organisms
☒	☐ Clinical data
☒	☐ Dual use research of concern
☒	☐ Plants

Methods

n/a	Involved in the study
☒	☐ ChIP-seq
☒	☐ Flow cytometry
☒	☐ MRI-based neuroimaging
